# Uptake of DNA by cancer cells without a transfection reagent

**DOI:** 10.1186/s40659-017-0107-x

**Published:** 2017-01-21

**Authors:** Yanping Kong, Xianbo Zhang, Yongliang Zhao, Yanfang Xue, Ye Zhang

**Affiliations:** 1 Department of Endocrinology, Dartmouth-Hitchcock Manchester, 100 Hitchcock Way, Manchester, New Hampshire 03104 USA; 2 Department of Surgery/Oncology, First Hospital of Shijiazhuang, 36 Fanxi Road, Shijiazhuang, 050011 Hebei China; 3 Laboratory of Disease Genomics and Individualized Medicine, Beijing Institute of Genomics, Chinese Academy of Sciences, 1 Beichen West Road, Beijing, 10029 China; 4 Department of Pharmacology, Hebei Medical University, 361 Zhongshan E Rd, Shijiazhuang, 050056 Hebei China; 5 National Laboratory of Medical Molecular Biology, Department of Biochemistry and Molecular Biology, Chinese Academy of Medical Sciences & Peking Union Medical College, Dongdan Santiao, Beijing, 100005 China

**Keywords:** DNA uptake, Cancer cells, Endocytosis

## Abstract

**Background:**

Cancer cells exhibit elevated levels of glucose uptake and may obtain pre-formed, diet-derived fatty acids from the bloodstream to boost their rapid growth; they may also use nucleic acid from their microenvironment. The study of processing nucleic acid by cancer cells will help improve the understanding of the metabolism of cancer. DNA is commonly packaged into a viral or lipid particle to be transferred into cells; this process is called transfection in laboratory. Cancer cells are known for having gene mutations and the evolving ability of endocytosis. Their uptake of DNAs might be different from normal cells; they may take in DNAs directly from the environment. In this report, we studied the uptake of DNAs in cancer cells without a transfection reagent.

**Methods:**

A group of DNA fragments were prepared with PCR and labeled with isotope phosphorous-32 to test their uptake by Huh 7 (liver cancer) and THLE3 (normal liver cells) after incubation overnight by counting radioactivity of the cells’ genomic DNA. Multiple cell lines including breast cancer and lung cancer were tested with the same method. DNA molecules were also labeled with fluorescence to test the location in the cells using a kit of “label it fluorescence in situ hybridization (FISH)” from Mirus (USA).

**Results:**

The data demonstrated that hepatocellular carcinoma cells possess the ability to take in large DNA fragments directly without a transfection reagent whereas normal liver cells cannot. Huh7 and MDA-MB231 cells displayed a significantly higher Rhodamine density in the cytoplasmic phagosomes and this suggests that the mechanism of uptake of large DNA by cancer cells is likely endocytosis. The efficacy of uptake is related to the DNA’s size. Some cell lines of lung cancer and breast cancer also showed similar uptake of DNA.

**Conclusions:**

In the present study, we have revealed the evidence that some cancer cells, but not nontumorigenic cells, can take DNA fragments directly from the environment without the aid of the transfecting reagent.

## Background

Cancer cells have been found to contain many gene mutations; they are malfunctioned in metabolism but they grow rapidly; their processing of nutrients and metabolism might be significantly different from normal cells. Tumor cells exhibit elevated levels of glucose uptake, a phenomenon that has been capitalized upon for the prognostic and diagnostic imaging of a wide range of cancers using radio-labeled glucose analogs [1]. In addition to synthesis, cancer cells may obtain pre-formed, diet-derived fatty acids by uptake from the bloodstream through lipoprotein lipase and fatty acid translocase, and this can fuel their growth [2]. Whether nucleic acid like DNA can be taken in by cancer cells from their microenvironment directly is unknown. DNA molecules are big in general; they may not enter normal cells freely. DNAs are normally packaged into viral or a lipid particle to be transferred into cells; this process is called transfection in laboratory. A reagent like FuGENE is commonly used for the purpose. Although our previous work demonstrated that packaged DNA fragments with lengths of 20–40 kb can recombine together by creating overlapping ends on HSV-1 DNA fragments in E Coli [3], whether living cells can take DNA fragments directly without a transfection reagent under a specific circumstance such as a cancerous condition is not clear. There are some previous reports suggesting direct uptake of large DNA by cells. When ^32^P-labeled liver chromatin was injected intravenously into a rat model with partial hepatectomy, radioactivity in the liver cells was found to be five times higher with ^32^P labeled chromatin than with inorganic ^32^P at 3 h post injection, implying that either whole chromatin or portions can be incorporated into nuclei of liver cells more rapidly than inorganic phosphate [4]. After naked vector DNA carrying the beta-glycosidase gene was injected into mouse muscles, the gene expression observed in muscle cells suggested that the cells may take in DNAs directly [5]. Malignant cells have unique metabolic features with completely different gene profiles compared with normal cells. They could acquire the ability to take in DNA fragments from the environment and use them for their fast growing needs. Here we provide our evaluation on the uptake of DNA in some malignant cells.

## Methods

### Cell lines

The THLE3 cells ordered from ATCC were SV40-large T immortalized human liver cells, which are nontumorigenic and AFP expression negative, and were cultured in BEBM medium (Lonza/Clonetics) supplemented with various growth factors. Liver cancer cell lines Huh7 and Hep3B (from ATCC) with a higher level of AFP expression were cultured in 1640 medium supplemented with 10% FBS. All other cell lines in the study were also from ATCC and cultured with the company’s protocols: HMEC (normal human mammary epithelial cells), NHBE (human bronchial epithelial cell), NMBC (nuclear matrix breast cancer), BEP2D (immortalized human bronchial epithelial cell), HM-hT (telomerase-immortalized human mammary epithelial cells), SW837 (adenocarcinoma, rectum), MB436 (adenocarcinoma, breast), MDA-MB231 (adenocarcinoma, breast epithelial), A549 (lung cancer), H1299 (lung cancer, epithelial), H460 (large cell lung cancer).

### DNAs and their labeling with α-^32^P-dCTP

The AFP DNA fragments with different lengths ranging from 57 to 1620 bp were prepared by PCR amplification in the presence of α-^32^P-dCTP (PerkinElmer) using AFP cDNA as template. The reaction was as follows: 10× LA buffer 5 µl; 10 mmol/l dNTP (dATP + dTTP + dGTP) 1 µl; 0.1 mmol/l dCTP 1 µl; sense primer 2 µl; antisense primer 2 µl; AFP template DNA (pCMV-sport6-AFP, 50 ng/µl) 1 µl; α-^32^P-dCTP 5 µl (10 µci/µl); LA Taq DNA polymerase 1 µl; H_2_O 32 µl. The PCR protocol: 94 °C 3 min; 94 °C 30 s, 55 °C 30 s, 72 °C 3 min for 40 cycles. The PCR products were then purified using G50 columns (Amersham) according to manufacturer’s protocol, and named as ISONA (isotope labeled nucleic acids). ISONA150 refers to a 150 base pair fragment; the primers for making this fragment are ACTCCAGTAAACCCTGGTGTTG and CTGAGCTTGGCACAGATCCTTATG. Genomic DNA isolated from Huh7 cells was ^32^P-labeled in the presence of α-^32^P-dCTP using a random labeling kit (New England Biolab following its protocol). The labeled products were purified by using G50 columns.

### Incubation of cells with α-^32^P-dCTP -labeled DNA fragments

The cells was seeded in 24 well plates with a cell density of 6 × 10^4^ cells per well. Twenty-four hours later, α-^32^P-dCTP -labeled DNA fragments were added into each well and the cells were kept in the incubator for different time periods as designed. Genomic DNAs were then isolated with DNAzole reagents, and radioactivity of genomic DNAs were quantified by a scintillation counter and visualized by dot-blotting after being exposed to Kodak films. The result of radioactivity was counted as counts per minute (CPM).

### Fluorescence labeling DNA to test its uptake in cancer cells and related normal cells

A kit of “label it fluorescence in situ hybridization (FISH)” from Mirus (USA) was used in this study following the company’s protocol. AFP cDNA (1.8 kb) was PCR-amplified, purified by phenol–chloroform extraction and adjusted to the concentration of 1 µg/µl. A 5 µg DNA template was used for fluorescein labeling at 37 °C for 1 h with Rhodamine, and the labeled probe was purified using a G50 column. The cells were incubated with the labeled probe at 37 °C for 16 h, then washed with PBS and fixed in 4% paraformaldehyde. After co-stained with DAPI, cell images were captured by a Leica confocal laser scanning microscope TCS SP (Leica). The fluorescence density of Rhodamine in cytoplasmic phagosomes was quantified by Image J software (http://imagej.net/Welcome). At least 100 cells were quantified for each cell line. The data in the bars represent mean ± SD from two independent experiments. Student’s t test was used for statistical analysis. p < 0.01 (**) was considered as statistically significant.

## Results

### DNA fragments were taken by HCC cells but not normal liver cells without transfection reagent

**Fig. 1:**
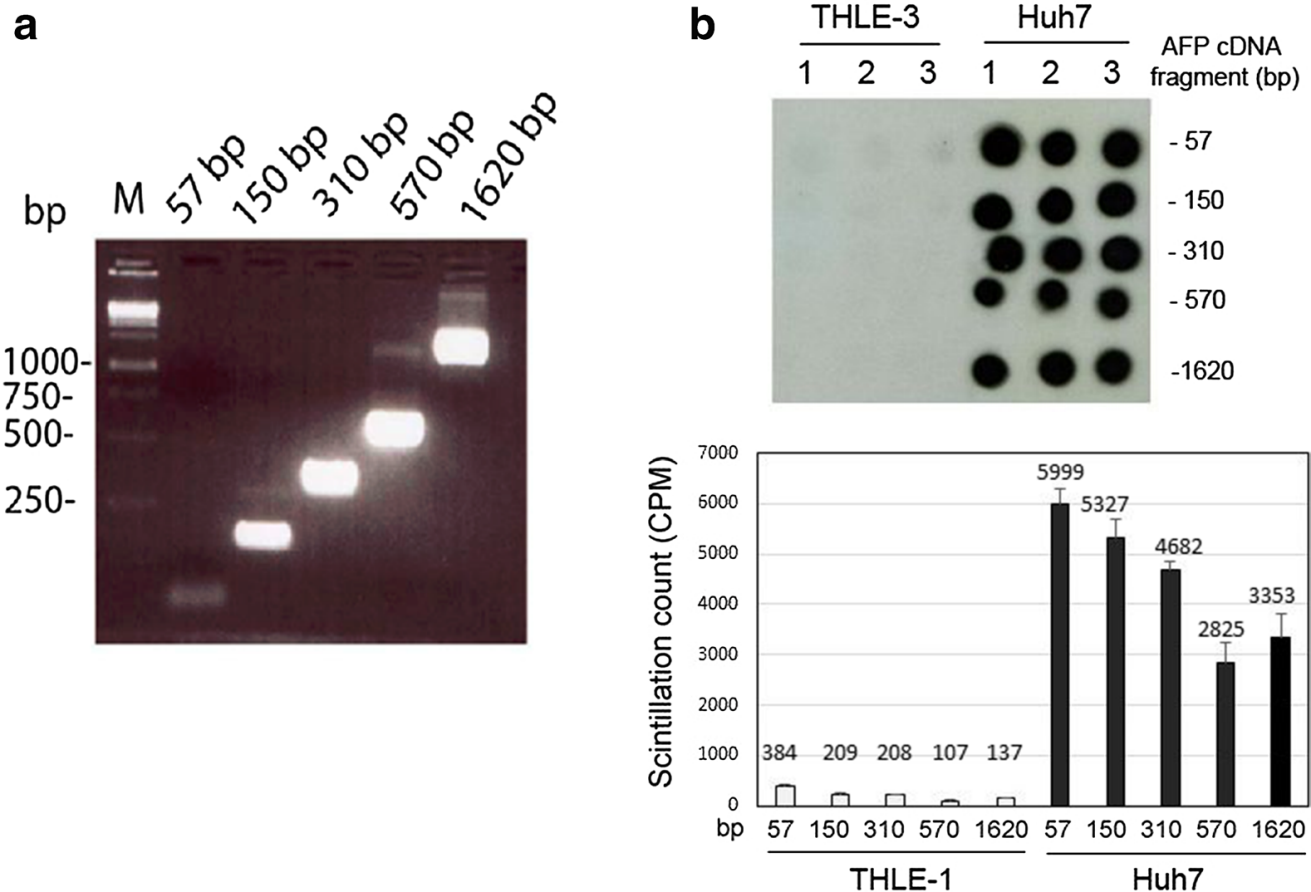
Uptake of DNA fragments with different size by HCC cells versus normal liver cells. **a** Different size of AFP cDNA fragments were prepared with PCR (57, 150, 310, 570 and 1620 bp). **b** After incubation with the cells overnight, radioactivity was visualized by dot blot (the *top picture*); triplicate wells were used to test each DNA fragment in the two cell lines. The radioactivity was also quantified by scintillation counter (the *lower picture*). The uptake is very strong in HCC cells but is minimal in normal liver cells. There is at least a tenfold difference

To prove the hypothesis that cancer cells may have the ability to take DNA fragments directly without packaging with transfection reagent, DNA fragments with different lengths were tested in THLE3 and Huh7 cells without adding the transfection reagent. As shown in Fig. 1a, the quality of a DNA fragment prepared with PCR was excellent. The radioactivity of genomic DNA isolated from AFP-positive Huh7 cells is significantly higher than that from AFP-negative noncancerous THLE3 cells. This data provided strong evidence that the naked DNA fragments without any packaging can be taken up by Huh7 cells (cancer), but not by THLE3 cells (normal). The ^32^P-labeled DNA had been integrated into genomic DNAs of Huh7 cells, and all sized DNA fragments in this experiment can enter into the cells with high efficiency even though the efficiency tends to be decreased along an increased length (Fig. 1b).

### Uptake efficiency of DNA fragment with or without a transfection agent

**Fig. 2:**
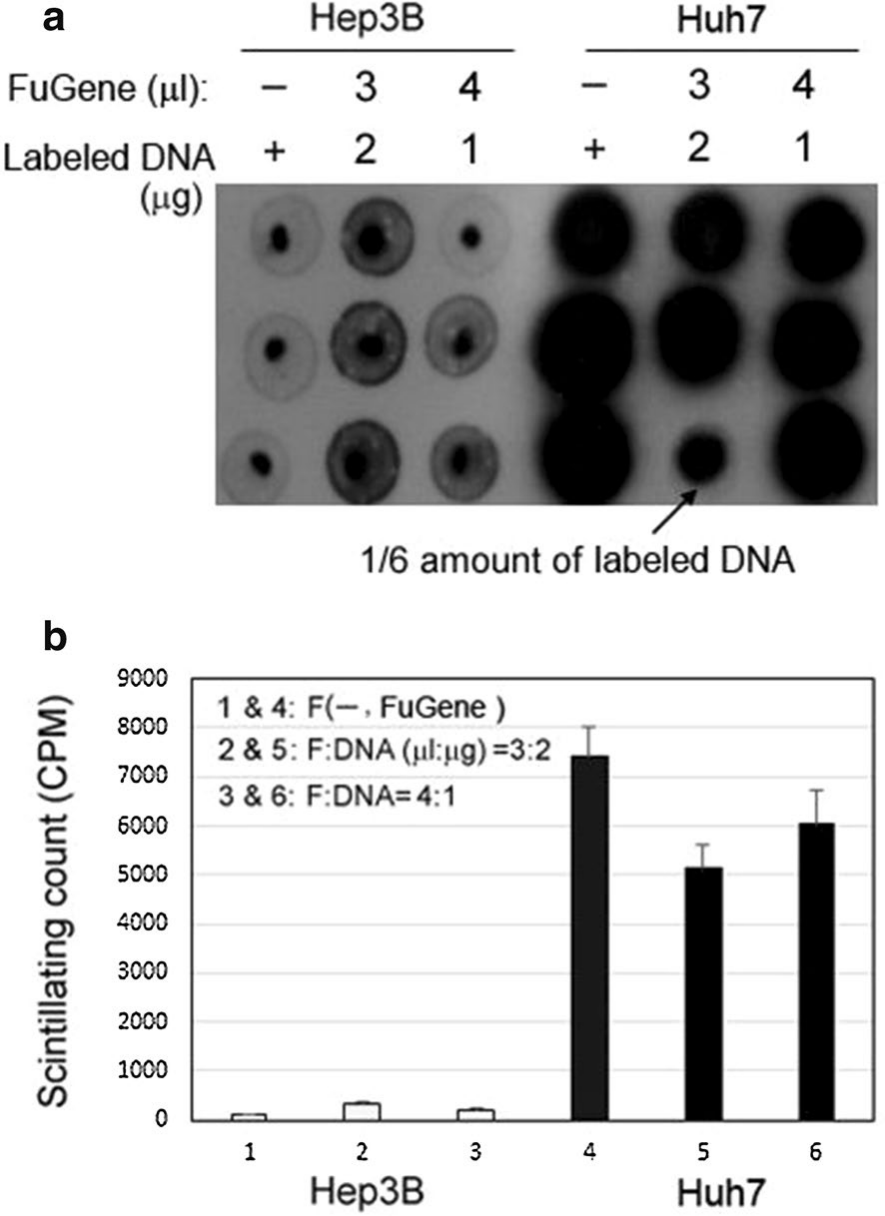
Uptake of ISONA by Huh7 and Hep3B cells with or without FuGENE The radioactivity was visualized by either dot blot (**a**) or quantified by scintillation counter (**b**). ISONA150 was added to Hep3B (l, 2, 3) and Huh7 [4–6]. *Lines 1* and *4*: 6 µl of 2 µg ISONA150 without FuGENE; *lines 2* and *5*: 6 µl of 2 µg ISONA150 + 3 µl FuGENE; *lines 3* and *6*: 6 µl of 1 µg ISONA150 + 4 µl FuGENE. In the third well of *line 5*: 1 µl of 0.33 µg ISONA + 4 µl FuGENE. *Line 4* of Huh 7 cells had strong uptake even without FuGene added. The well of *line 5* with less DNA probe showed much lower uptake compared with others of *4*, *5* and *6*

ISONA150 was tested in two kinds of liver cancer cell lines—Huh7 and Hep3b with or without FuGENE (Fig. 2). FuGENE is commonly used in labs to facilitate a cell’s uptake of DNAs. In Huh7, cells with more DNA added have higher radioactivity and there was no significant difference with a different concentration of FuGENE. The data here suggested that uptake of ISONA is independent of FuGENE but related to the amount of DNA. In Hep3b cells, the uptake is much lower compared with Huh7.

### Uptake of ISONA150 compared with genomic DNAs; DNA size and uptake; and uptake of ISONA in different cell lines

**Fig. 3:**
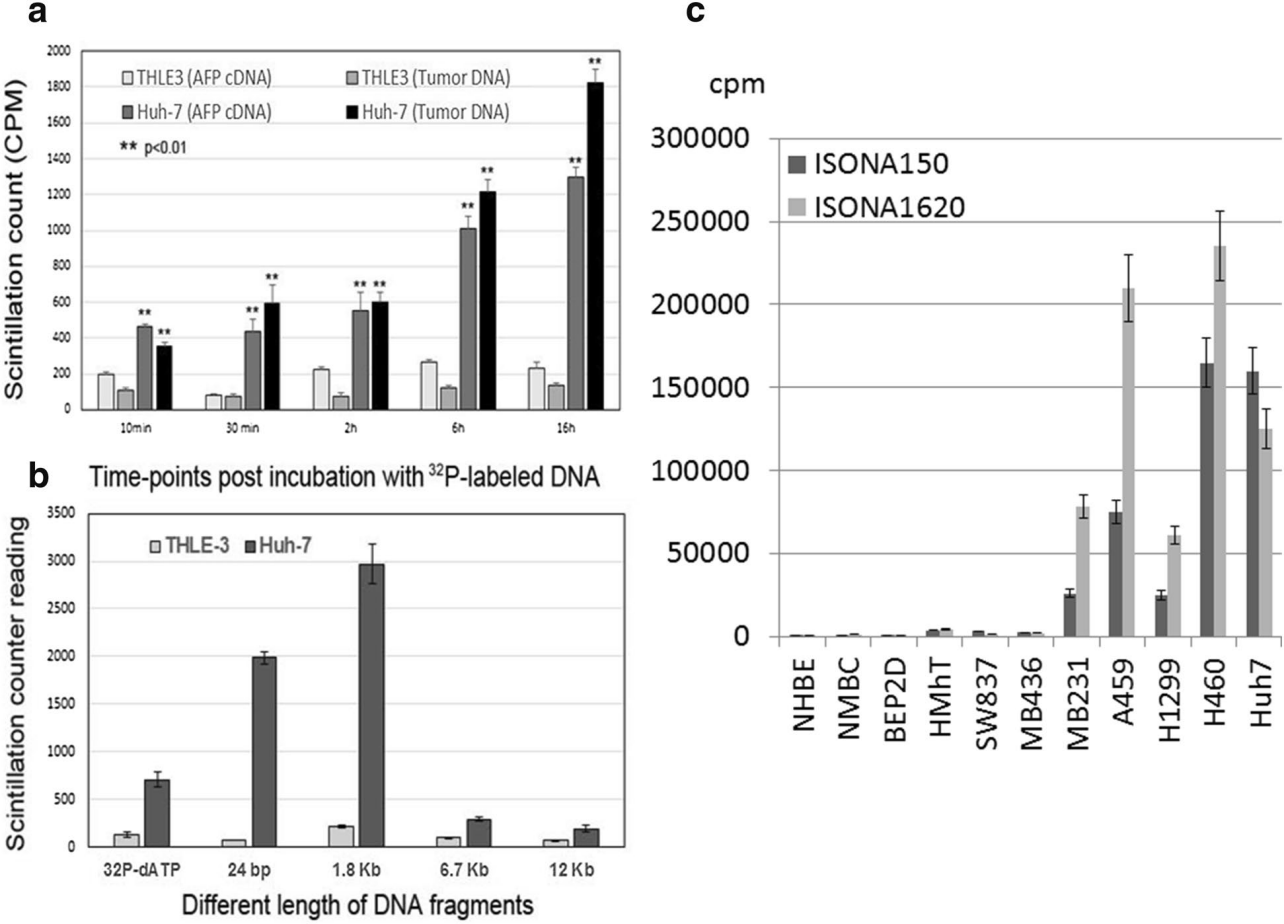
Uptake of AFP DNA or genomic DNA by HCC cells, and uptake of ISONA by different cell liners. **a** Both ISONA150 and genomic DNAs can be taken up efficiently by Huh7 but not by THLE-3 cells. The uptake was significant as early as 10 min after DNA was added. **b** Uptake of DNA fragments was compared with single nucleotide, it is size dependent and unlikely to be taken up when the size is over 6.7 kb. **c** ISONA compounds were incubated with different cell lines overnight, uptake was significant in breast cancer, lung cancer and liver cancer cells. The uptake is minimal in noncancerous cells

Uptake of ISONA150 and ^32^P-dCTP-labeled genomic DNA from Huh-7 cells by the random labeling kit was tested in Huh7 cells at different times of incubation. The efficiency of uptake of both DNA fragments as shown in Fig. 3a, was comparably similar, but it is more efficient with the genomic DNA. DNA fragments with designated sizes were absorbed into Huh7 cells much more efficiently than a single nucleotide was and this was related to DNA size (Fig. 3b). We further tested the uptake efficiency of ISONA by different histological types of human cancer cells with undetectable levels of AFP expression. Intriguingly, some breast cancer and lung cancer cell lines displayed a very high level of uptake; whereas, nontumorigenic cells like BEP2D showed a very low level of uptake (Fig. 3c). The test results suggest that only cancer cells evolved the ability to directly take up large DNA fragments. Moreover, selectivity for the types of DNA in the uptake by different cancer cells was not observed.

### Fluorescence labelled DNA uptake

**Fig. 4:**
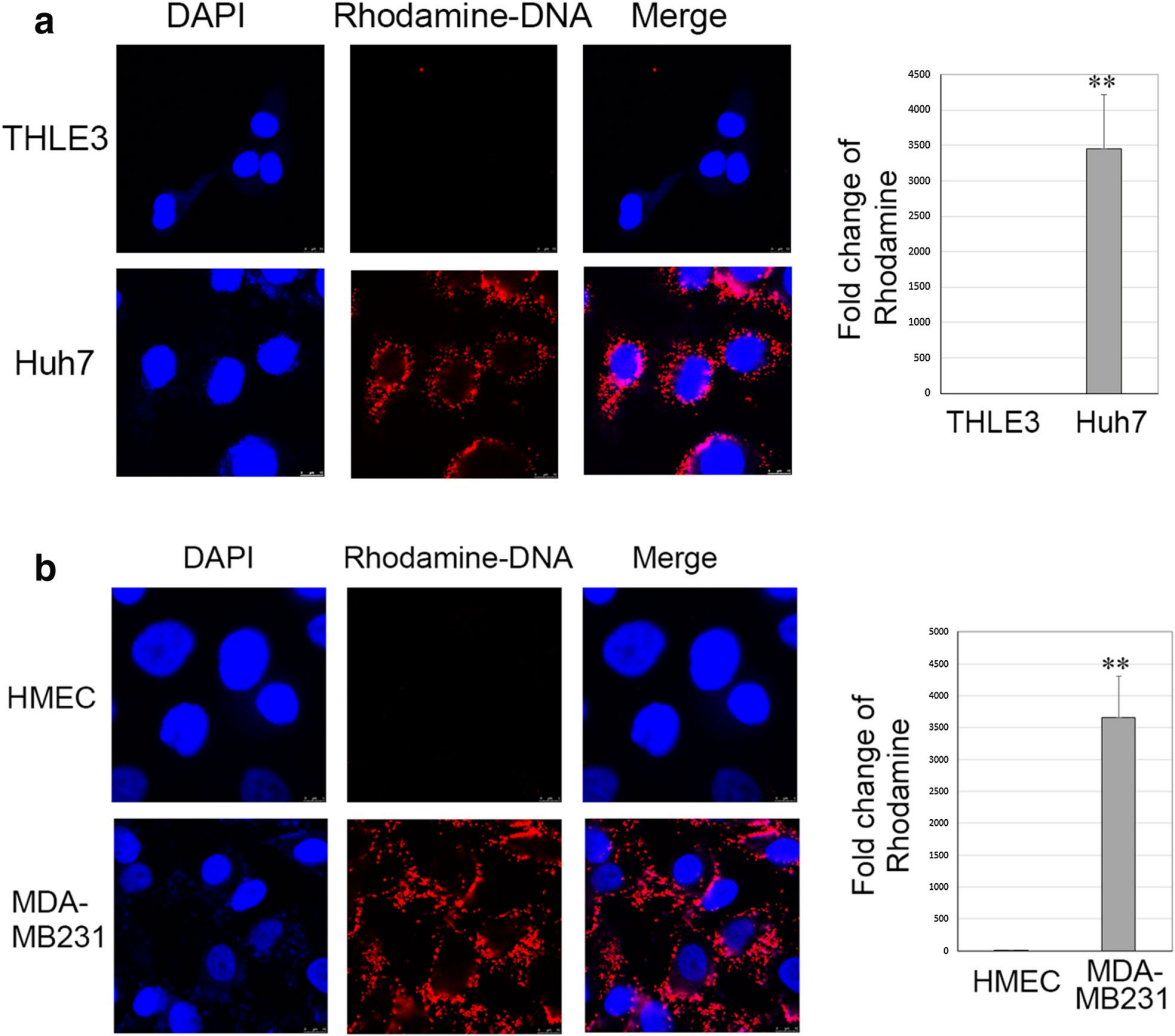
Fluorescence study of DNA uptake by cancer cells and their corresponding normal cells. A Rhodamine labeled AFP cDNA fragment (1.8 kb) was tested in Huh7 (**a**) and MDA-MB231 (**b**); also in their corresponding normal cells of THEL3 and HMEC. Huh7 and MDA-MB231 cells displayed a significant higher Rhodamine fluorescence density in the cytoplasmic phagosomes; but the normal cells did not. Intriguingly, some of the phagosomes can be counterstained by DAPI

Uptake of Rhodamine fluorescence labeled DNA probe was further tested in the normal and cancer cells, and analyzed under confocal microscope. As shown in Fig. 4, after overnight incubation with fluorescence-labeled DNA probe, a significantly higher level of Rhodamine fluorescence was observed in the cytoplasm of cancer cells (Huh7 and MDA-MB231), but not in their corresponding normal control cells (THEL3 and HMEC). In addition, the Rhodamine fluorescence was mainly localized in the phagosomes of cytoplasmic compartment. Intriguingly, some of the phagosomes can be counterstained by DAPI, demonstrating that rhodamine-labeled DNA has truly entered into the cells by phagocytosis.

## Discussion

DNA transfer into cells with a transfection reagent has been well studied for the purpose of gene therapy [6, 7]. How cancer cells process the uptake of nucleic acids in a biological environment is not clear. Investigation of nucleic acid uptake by cancer cells will help improve the understanding of the metabolism of cancer. This study provides evidence that cancer cells including HCC but not normal liver cells can take large naked DNA fragments (compared with oligonucleotides which are normally less than 20 base pairs and considered small) without the aid of either viral proteins or liposome/FuGENE reagents. AFP DNA was chosen in this study because it is unique in liver cancer and was considered as highly needed by the cells. Huh7 cells that express a high level of endogenous AFP gene revealed a high capacity of uptake of AFP DNA fragment. This leads to a possible explanation that the uptake of DNA by cancer cells might be need-based. However, lung cancer and breast cancer cells can also take AFP DNA the same way; they might digest the DNAs first and then have those nucleotides used. The detailed mechanism of DNA fragments transportation into cells is still not clear. To determine whether endocytosis may serve as the possible mechanism for cellular DNA transportation, a fluorescence study was carried out to test the phagocytosis in HCC and breast cancer cells in comparison with their corresponding normal cells following the recommended protocol [8]. Since FITC (Fluorescein Isothiocyanate) fluorescence dye is pH-sensitive and can be quenched within phagosomes with acidic condition, we used rhodamine dye exhibiting much less quenching over a wide range of pH (from pH 4 to 9) to label the AFP cDNA (1.8 kb) using *label* IT Nucleic Acid labeling kit. The results showed that Huh7 and MDA-MB231 cells displayed a significantly higher Rhodamine density in the cytoplasmic phagosomes. Intriguingly, some of the phagosomes can be counterstained by DAPI, demonstrating that rhodamine-labeled DNA has truly entered into the cells by phagocytosis. Glucose and amino acids are transported through the cell membrane by specific transporters located on the cell membrane. At least four glucose transporters have been identified so far through the studies of glucose metabolism in diabetes [9, 10]. The small DNA fragment may go through a similar pathway. Mellman and Yarden [11] wrote a review in 2013 describing endocytosis of cancer cells, in which it stated that cancer cells might be able to undergo endocytosis to match their high proliferation pace. The endocytosis is possibly related to gene mutations like P-53 [12]. Some well-known proteins from cancer were found responsible for the processing of endocytosis and related to the cancers’ aggressiveness [13]. Malignant cells with a high proliferation rate might evolve a specific mechanism for utilizing the extracellular DNA fragments to support their high rate of DNA replication. Our study using fluorescent-labeled DNA fragment revealed that the DNAs were taken up by phagocytosis. This suggests that the uptake of larger DNA by cancer cells is likely through endocytosis. The fact that the uptake of DNA is not mainly dependent on the transfection agent FuGENE also suggests that it is not utilizing passive transporting. Large DNA was absorbed into Huh7 cells much more efficiently than a single nucleotide and was size sensitive as shown in Fig. 3b. All the DNA fragments used in the test were from AFP genomic DNA; the result might be secondary to the efficacy of endocytosis which may be related to the molecule’s size. The study of different cell lines also revealed that those aggressive cancers have the ability to take large DNA, which supports the hypothesis that endocytosis is related to the aggressiveness of cancer. Meanwhile, the uptake study showed no uptake of the DNA fragments by normal liver cells and breast cells. This reveals that endocytosis of DNA is unlikely to exist in those cells. The difference might be used for cancer treatment. The uptake happens as early as 10 min after DNA is added to the cell culture; it demonstrates that the cancer cell can take those DNAs very efficiently. As for some cancer cells like Hep3B that did not show the same ability to take those DNA fragments as Huh7, further investigation is required. As mentioned above, the endocytosis of cancer cells are related to some genes mutations especially p-53. Hep3B has no expression of p-53, but Huh7 has mutated p-53 [14]; this might cause the difference in the ability of cellular endocytosis. The detailed mechanism of exogenous DNA transporting into cancer cells worth further evaluation.

## Conclusions

In conclusion, our study suggests that cancer cells may take in large DNA directly from the environment and that is possibly through endocytosis which does not happen in normal cells.

## Abbreviations

HCC: hepatocellular carcinoma
ISONA: isotope labelled nucleic acid
